# Kawasaki disease and hepatobiliary involvement: report of two cases

**DOI:** 10.1186/s13052-016-0238-7

**Published:** 2016-03-08

**Authors:** Ingeborg Marianne Keeling, Elisabeth Beran, Otto Eugen Dapunt

**Affiliations:** Department Cardiac Surgery, Medical University of Graz, Auenbruggerplatz 29, 8036 Graz, Austria

**Keywords:** Kawasaki disease, Hepatopathy, Cholestasis, Ischemic optic nerve neuropathy, Differential diagnosis

## Abstract

**Background:**

Kawasaki disease (KD) without affection of the coronary artery system is rare. Optic nerve pathology together with KD has not been described earlier.

**Case presentation:**

We present one case of KD in a 12-year-old girl predominantly with prolonged cholestasis, and a second case of multiple recurrent KD in a 9-year-old boy with hepatomegaly and ischemic optic nerve neuropathy. The coronary artery system was not involved in either case.

**Conclusions:**

KD warrants rapid diagnosis and immediate specific treatment in order to prevent the high risk of coronary artery aneurysm and stenosis.

## Background

Various manifestations of Kawasaki disease (KD) occur at an early age. When the hepatobiliary tract is primarily affected, the diagnosis relies mainly on symptoms, ultrasonography and laboratory parameters of systemic vasculitis. Numerous diseases must be excluded. Incomplete and atypical cases are difficult to detect. Rapid diagnosis and treatment are crucial, as 4 % of KD patients develop coronary artery (CA) involvement [1].

## Case presentation

### Case 1

A 12-year-old girl presented with a 6-day history of subfebrile temperature, generalized lymphadenitis, fatigue and inappetence. Exanthema of the extremities and trunk, raspberry tongue, bulbar injection, facial edema, palmar erythema, swelling of the small-finger joints, discrete jaundice and epigastric tenderness were observed. The general practitioner (GP) had treated her (benzathin-phenoxymethyl penicillin, 750 mg TID) for suspected scarlet fever.

Laboratory data (and normal range in parenthesis) were: WBCC 15 400/mm^3^ (4 500–12 000/mm^3^), PMN cells 73.4 % (50.0–75.0 %), lymphocytes 9.4 % (20.0–40.0 %), eosinophils 9.0 % (0–5.0 %), platelets 451 000/mm^3^ (140 000–440 000/mm^3^), CRP 15 mg/l (0–8 mg/l), ASL 971U/ml (0–125U/ml), total bilirubin 5.37 mg/dl (0–1.30 mg/dl), esterized bilirubin 2.86 mg/dl (<0.20 mg/dl), ASAT 17U/l (0–21U/l), ALAT 42U/l (0–22U/l), GGT 50U/l (0–19U/l), AP 557U/l (0–390U/l), HDL cholesterol 5 mg/dl (≥37 mg/dl), bilirubinuria, and ketonuria. Hepatitis screening revealed borderline complement binding reaction for Enterovirus and Coxsackievirus types other than A9. The chest x-ray showed prominent hila and the ECG a normofrequent sinus rhythm. Echocardiography indicated normal left ventricular function. CAs had normal dimension, no signs of aneurysm. Liver ultrasonography revealed distinct periportal fields. Clinical symptoms suggested KD with uncommon cholestasis. On the day of admission, treatment was started with intravenous immune globulin (IVIG) (Octagam®, 1.6 g/kg/d), aspirin (ASA, 500 mg TID), sucralfate (1 g TID), and ursodeoxycholic acid (100 mg TID).

Desquamation of the fingertips and pronounced jaundice developed. The blunt liver margin was palpated 0.5–1 cm underneath the right costal edge and hepatic consistency was found normal. Moderately acholic stools and hyperchromic urines were noted. Ultrasonography and MRI showed hepatomegaly (13x16x9.6 cm) and regular width of intra- and extrahepatic bile ducts. After 10 days, the patient improved and was discharged with ASA treatment.

Immunologic screening revealed positive anti-Ro-antibodies and a positive anti-U_1_-snRNP subset (against extractable nuclear antigen-ribonucleoprotein). Four months after discharge, transaminases and platelet counts normalized while total cholesterol (249 mg/dl, normal <200 mg/dl) and eosinophils (11.1 %, normal 0–5.0 %) remained elevated.

### Case 2

Since his fourth month of life, a 9-year-old boy experienced upper respiratory infections and recurrent otitis.

At the age of 4 years he was admitted 5 days after headaches, conjunctival injection, fever, fatigue, enanthema of the throat and tonsils, raspberry tongue and enlargement of submandibular lymph nodes evolved. Laboratory results were: WBCC 27 300/mm^3^, (4 500–12 000/mm^3^), platelets 594 999/mmm^3^ (140 000–440 000/mm^3^), CRP 25 mg/l (0–8 mg/l), ESR 60/96 (3–13 mm/1 h), ASL and RF negative. Although an exanthema was missing, clinical findings suggested incomplete KD. Treatment consisted of IVIG (Octagam®, 2 g/kg/d), and high-dose ASA (300 mg QID) for 3 weeks. Fever resolved within 24 h. Desquamation of the toes developed upon discharge.

After persistent fever for 10 days at age 6 years, resistant to antibiotic treatment occurred along with repeated vomiting, fatigue, angina, headaches, photophobia, raspberry tongue and desquamation of the toes. Laboratory data were: WBCC 21 800/mm^3^, platelets 746 000/mm^3^, CRP 22 mg/l, ESR 110 mm/1 h. Recurrent incomplete KD was diagnosed. The child developed intolerance to IVIG (Endobulin®, 5 g), with severe abdominal pains. The liver margin was palpable 0.5 cm below the right costal edge, hepatic consistency was normal. Stool and urine were unremarkable. Only once during the hospital stay ASAT 26U/l (0–21U/l) and ALAT 23U/l (0–22U/l) were slightly elevated. CT showed hepatomegaly, a prominent, homogenous pancreas, and reflections, possibly small sedimentations, in the choledochus duct. The examination neither confirmed nor dismissed an autoimmune disease. Reduced tissue necrosis factor (TNF)–receptor levels (0.81 ng/ml; 50 % of the minimum norm) suggested autosomal-dominant TNF-receptor associated periodic fever syndrome (TRAPS), but exon 2–5 mutations of the TNFR1A1 gene were not found in PCR.

At the age of 9 years the patient was admitted for acute right-sided ablepsia. The ophthalmologist diagnosed ischemic optic nerve neuropathy. High-dose steroids for 13 days incompletely restored the child’s vision. The examination for an autoimmunologic disease was negative.

## Conclusions

In both exposed cases, KD was strongly suggested at admission. Uncommon clinical features in these two children were hepatopathy, cholestasis and diarrhea. In case 1, hepatomegaly and cholestasis, with distinct periportal fields, were identified by ultrasonography and MRI. In case 2, hepatomegaly with cholangitis was present. The CA system was unaffected. The predominant gastrointestinal manifestation in up to 13.9 % of KD cases is acalculous gallbladder hydrops, which may require cholecystectomy or percutaneous transhepatic biliary drainage [2]. Hepatopathy exceptionally rarely induces hepatocellular necrosis [3]. Other manifestations include hepatitis, pancreatitis, enteritis and ileus. Gilbert’s syndrome with concurrent infection may resemble KD in clinical terms.

Childhood diseases like scarlet fever may manifest symptoms of hepatopathy and cholestasis. Coxsackievirus and Enterovirus associated hepatopathy were unlikely, as cholestasis without hepatitis, and elevated ESR and PMN are usually unrelated to these viral infections. In any case, KD-related macrophage activation syndrome (MAS) should be also suspected if liver dysfunction presents with other typical abnormal laboratory findings, such as cytopenia, hyperferritinemia and elevated serum LDH.

Initial antibiotic treatment for suspected bacterial infection, as in the present case, renders drug side-effects an important differential diagnosis. In case 1, where positive Ro-antibodies, extractable nuclear antigen-ribonucleoprotein (U_1_-snRNP), and elevated eosinophils suggested autoimmune hepatopathy rather than penicillin reaction, jaundice subsided without treatment 5 weeks post-discharge.

Toxic shock syndrome (TSS) may mimic KD. A connection between staphylococcal and streptococcal TTS and KD has also been suggested [1].

Specific immunologic alterations may be found in KD, which requires extensive immunologic testing and the use of image processing to confirm KD diagnosis. Therefore, in complex KD cases multispecialist approach, including the pediatric cardiologist, immunologists, rheumatologist, hepatologist, infectiologist, ophthalmologist, intensive care personnel, and clinical pharmacologist, are essential to avoid the many pitfalls of rapid diagnosis.

KD patients with fever, CRP >10 mg/dl, LDH >590U/l and/or hemoglobin <10 g/dl, and those with high bilirubin and transaminases, are considered non-responsive to IVIG [4]. IVIG resistance with persistent or recrudescent fever occurs in approximately 18 % of KD patients. Retreatment involved more individual adjustment, consisting of intravenous steroid pulse therapy, infliximab, cyclophosphamide, anakinra, etanercept, methotrexate, or plasmapheresis [1, 4–6]. Additionally, antioxidants, as vitamin C, and drugs affecting cholesterol levels, can reduce endothelial dysfunction.

Abnormalities of liver function tests are frequently found in patients with acute KD. Indeed, children with abnormal liver function tests are at higher risk for IVIG resistance [7, 8]. Moreover, ultrasonographic biliary findings or higher AST levels may be risk factors for coronary artery abnormality as a complication and/or recurrent KD if they are present during the first episode [9, 10].

After repeated KD episodes, case 2 developed an extremely rare ophthalmologic KD manifestation, specifically ischemic optic nerve neuropathy, which to our knowledge is the first reported case of optic nerve pathology associated with KD [11]. However, transient affliction of the cranial nerves may at times be either the presenting feature or a complication of an otherwise uncomplicated KD in infants and children. Oculomotor nerve palsy may resolve after IVIG therapy [12].

In conclusion, atypical KD calls for extreme alertness, rapid diagnosis and immediate treatment if suggested. The absence of CA lesions and the presence of cholestasis may delay the diagnosis and specific treatment of this potentially life-threatening disease with severe long-term consequences.

### Consent

Written informed consent was obtained from the parents of the patient for publication of this case report. A copy of the written consent is available for review by the Editor-in-Chief of this journal.

## Abbreviations

GP: general practitioner
TID: three times per day
QID: four times per day
ASL titer: antistreptolysine titer
WBCC: white blood cell count
PMN: polymorphonuclear
CRP: C-reactive protein
ESR: blood sedimentation rate
ATIII: antithrombin III
ASAT: aspartate amino transferase
ALAT: alanine amino transferase
GGT: γ-glutamyl transferase
AP: alkaline phosphatase
HDL: high-density lipoprotein
RBCC: red blood cell count
MRI: magnetic resonance imaging
